# Traumatic Brain Injury Precipitates Cognitive Impairment and Extracellular Aβ Aggregation in Alzheimer's Disease Transgenic Mice

**DOI:** 10.1371/journal.pone.0078851

**Published:** 2013-11-04

**Authors:** Naoki Tajiri, S. Leilani Kellogg, Toru Shimizu, Gary W. Arendash, Cesar V. Borlongan

**Affiliations:** 1 Center of Excellence for Aging and Brain Repair, Department of Neurosurgery and Brain Repair, University of South Florida Morsani College of Medicine, Tampa, Florida, United States of America; 2 Department of Psychology, University of South Florida, Tampa, Florida, United States of America; 3 NeuroEM Therapeutics, Inc., Scottsdale, Arizona, United States of America; National Center for Geriatrics and Gerontology, Japan

## Abstract

Traumatic brain injury (TBI) has become a signature wound of the wars in Iraq and Afghanistan. Many American soldiers, even those undiagnosed but likely suffering from mild TBI, display Alzheimer's disease (AD)-like cognitive impairments, suggesting a pathological overlap between TBI and AD. This study examined the cognitive and neurohistological effects of TBI in presymptomatic APP/PS1 AD-transgenic mice. AD mice and non-transgenic (NT) mice received an experimental TBI on the right parietal cortex using the controlled cortical impact model. Animals were trained in a water maze task for spatial memory before TBI, and then reevaluated in the same task at two and six weeks post-TBI. The results showed that AD mice with TBI made significantly more errors in the task than AD mice without TBI and NT mice regardless of TBI. A separate group of AD mice and NT mice were evaluated neurohistologically at six weeks after TBI. The number of extracellular beta-amyloid (Aβ)-deposits significantly increased by at least one fold in the cortex of AD mice that received TBI compared to the NT mice that received TBI or the AD and NT mice that underwent sham surgery. A significant decrease in MAP2 positive cells, indicating neuronal loss, was observed in the cortex of both the AD and NT mice that received TBI compared to the AD and NT mice subjected to sham surgery. Similar changes in extracellular Aβ deposits and MAP2 positive cells were also seen in the hippocampus. These results demonstrate for the first time that TBI precipitates cognitive impairment in presymptomatic AD mice, while also confirming extracellular Aβ deposits following TBI. The recognition of this pathological link between TBI and AD should aid in developing novel treatments directed at abrogating cellular injury and extracellular Aβ deposition in the brain.

## Introduction

Traumatic brain injury (TBI) is the result of an acute insult to the head due to a variety of external causes, such as a motor vehicle accident, firearm, or fall. Interestingly, initial trauma to the head is often followed by secondary brain tissue damage, which is recognized as a major risk factor for Alzheimer's Disease (AD) [1]–[5]. The Centers for Disease Control and Prevention estimates that over 5.3 million Americans live with disabilities as a result of TBI [6], [7]. Despite the staggering number, the consequential biological processes accompanying TBI are poorly understood. It is critical that we develop strategies to limit the secondary brain damage that follows initial head trauma and to devise effective therapies to improve long-term recovery of function. AD is a neurodegenerative disorder characterized pathologically by progressive neuronal loss and both intraneuronal and extracellular aggregates of beta-amyloid (Aβ) peptides. Although a pathological link between TBI and AD has not been well-defined, a tau pathology referred to as chronic traumatic encephalopathy (also called dementia pugilistica) has been described in the brains of individuals exposed to repetitive, often mild or concussive, head injury such as boxers [8], [9] and football players [10]–[13]. This study is designed to clarify the pathological link between TBI and AD. We hypothesized that TBI precipitates early presence of AD, with pathological disturbance manifested in discreet brain regions (such as the cortex and the hippocampus), along with accompanying cognitive impairment [14], [15]. While the cortex is directly impacted in TBI, secondary cell death ensues in the hippocampus, a critical brain structure for memory and learning. Indeed, many patients later suffer from TBI-related dementia, implicating the role of the hippocampus in the disease progression [16], [17]. Because the adult hippocampus is a well-established neurogenic site highly sensitive to both acute and chronic injury [18]–[20], this specific brain region appears to be an optimal candidate to study secondary pathological disturbance after an initial cortical injury. Interestingly, accelerated Aβ aggregation has been recently detected in transgenic AD mice at 24 hours and seven days after TBI [21]. Moreover, the systemic treatment of a compound designed to inhibit γ-secretase activity, a proteolytic process required for Aβ production, suppressed the TBI-induced Aβ accumulation in these injured mice at both time points [21]. Extending this acute TBI-induced AD histopathology to a more long-term period, and demonstrating its behavioral correlate (specifically an expedited cognitive decline), should further advance the TBI and AD pathological link.

Here, we examined the possible exacerbating cognitive and histopathological impact of TBI on AD in presymtomatic APP/PS-1 AD-transgenic mice and non-transgenic (NT) mice using the controlled cortical impact (CCI) model. The present study began at age three months and ended at age five months. This study should provide a better understanding of the long-term role of trauma in AD pathology, thereby advancing novel treatments aimed at arresting cellular injury and removing Aβ deposition from the brain to retard the symptoms of AD and TBI.

## Materials and Methods

### Ethics Statement

All experiments were conducted in accordance with the recommendations in the Guide for the Care and Use of Laboratory Animals of the National Institutes of Health. The protocol was approved by the Institutional Animal Care and Use Committee at the University of South Florida. All efforts were made to minimize animal suffering. Mice were housed individually, in a temperature- and humidity-controlled room that was maintained on 12/12 hour-light/dark cycles. They had free access to food and water.

### Subjects

The subjects were obtained through pre-determined breeder pairs, combining mutant Tg2576 APP mice with mutant PS1 line 5.1 transgenic mice, which generated NT, APP, APP+PS1, and PS1 transgenic offspring with a mixed background of (C57B6/SJL/SwissWebster/B6D2F1). Upon weaning, subjects were genotyped by Southern blot analysis. Three-month old AD transgenic mice were used in this study. This age represents the AD pre-symptomatic stage in this particular transgenic mouse, wherein mice are cognitively normal although they have already begun to produce and accumulate Aβ in their brains. To clarify the effects of TBI on these mice, cognitive and neurohistological effects were evaluated for two different cohorts of animals. The first cohort included four treatment groups: AD mice + TBI (AD-TBI; n = 7), AD mice + sham surgery (AD-Sham; n = 8), NT mice + TBI (NT-TBI; n = 10), and NT mice + sham surgery (NT-Sham; n = 9). The second cohort included the same four treatment groups: AD-TBI (n = 8), AD-Sham (n = 8), NT-TBI (n = 7), and NT-Sham (n = 7). Animals in cohort 2 were exposed to the same surgical procedure, but were not subjected to any behavioral testing before and after the surgery.

### Pre-surgery Procedures

To assess cognitive changes following TBI, all mice in cohort 1 were subjected to 14 days of pre-TBI training in the radial arm water maze (RAWM) task. The apparatus was a black circular pool (100 cm diameter), in which an aluminum insert was placed to form a RAWM. In the apparatus, six swim arms (32 cm length and 19 cm width) radiated from the central circular swim region (36 cm diameter). A clear round platform (9 cm diameter) was placed near the end of one of the arms at 1.5 cm beneath the surface of the water. Surrounding the pool, a visual cue was placed at each end of the radial arms, including an inflatable flamingo, a bouquet of silk flowers, a bean bag pumpkin, a plush flower, and a beach ball. These visual cues were placed at random heights and proximity to the pool and aligned with the center of each arm. Additional extramaze cues (e.g. light fixtures, cabinets, etc.) were also present, with the experimenter remaining stationary at the same location to also serve as one of the visual cues. Each daily session consisted of four acquisition trials (T1–T4), followed by a 30-minute delay interval and then a retention trial (T5). For each session, the escape platform was placed at the end of one of the goal arms, with a different semi-randomized goal arm selected for each daily session. At the beginning of each trial, the subject was placed at the end of one of the remaining five non-goal arms. The mouse was positioned facing the wall, away from the center. Each trial lasted 60 seconds, during which an animal was allowed to swim in order to find the platform. The number of entries to the non-goal arms were recorded as errors. The error occurred when the subject's full body length entered into an incorrect arm, including the goal arm if the platform was not found. The subject was allowed to swim to the end of the incorrect arm, then gently guided back to the starting location after every error. Once the subject found the platform, a 30-second resting period on the platform was permitted before beginning the next trial for T1-T4. If the subject failed to find the platform within 60 seconds, it was guided to the platform and allowed a 30-second resting period. After the completion of T4, the mouse was removed from the pool, dried with a towel, and returned to its cage for 30 minutes. The procedures for T5 were identical to T1-T4, with the start arm being the one remaining non-goal arm not used in the four prior trials for that day. Only one five-trial session per day was performed. Pre-TBI testing sessions continued for a total of 14 days until all mice performed at an average of two or fewer errors across T4 and T5. The experimenter remained unaware of the animals' genotypes throughout all of the behavioral testing. After training, there were no performance differences between AD mice (n = 19) and NT mice (n = 19), thus ten of the AD mice and ten of the NT mice received an experimental TBI, while the remaining nine mice in each of the AD and NT groups received sham surgery. At two and six weeks after surgery, all mice were tested in the same water maze task.

### Surgical Procedures

Presymptomatic AD and NT mice were subjected to unilateral moderate TBI using the CCI model (Pittsburgh Precision Instruments, Inc, USA). Animals were deeply anesthetized using the intraperitoneal injection of a combination of ketamine (100 mg/Kg) and xylazine (10 mg/Kg). Once deep anesthesia was achieved, individual animals were fixed in a stereotaxic frame (David Kopf Instruments, Tujunga, CA, USA). After exposing the skull, a 4.0 mm craniectomy was performed over the right frontoparietal cortex (**−**2.0 mm anteroposterior and +2.0 mm mediolateral to bregma) [22]. The pneumatically operated TBI device impacted the brain at a velocity of 6.0 m/s reaching a depth of 1.0 mm below the dura mater layer, and impacted the brain for 150 ms. The impactor rod was angled 15° to the vertical to maintain a perpendicular position in reference to the tangential plane of the brain curvature at the impact surface [23]. A linear variable displacement transducer (Macrosensors, Pennsauken, NJ) measured impact velocity and duration. Mortality associated with this experimental TBI was about 5%, with no significant differences between AD and NT mice.

### Post-surgery Procedures

**Figure 1:**
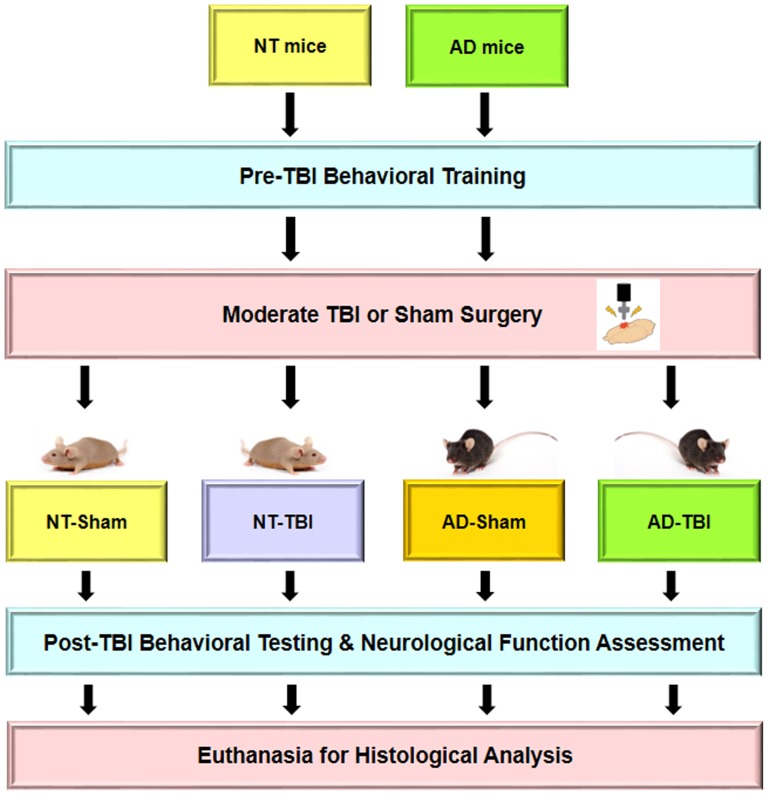
Experimental design is shown. For cohort 1, animals were trained in the RAWM prior to TBI surgery and then tested for cognitive deficits in the RAWM at two and six weeks post-TBI. For cohort 2, there was no pre-TBI behavioral training or post-TBI behavioral testing. All the animals were euthanized at six weeks post-TBI and the brains were harvested for histological analysis.

Post-TBI behavioral testing sessions for cohort 1 were conducted two and six weeks after the TBI procedure. The procedures were the same as the pre-TBI training, except that the post-TBI testing consisted of four consecutive sessions (days) regardless of the behavioral performance. Directly following the completion of the final post-TBI testing session, neurological functioning for all groups was assessed using balance beam and visual cliff tasks [24]. A schematic diagram of experimental design is shown (Figure 1).

### Fixation and Sectioning

For cohort 1, at six weeks post-TBI, mice were deeply anesthetized with 1–2% ketamine and xylazine and perfused from the ascending aorta with 20 mL of cold phosphate buffer saline (PBS) followed by 20 mL of 4% paraformaldehyde in phosphate buffer (PB). Brains were removed and post-fixed in the same fixative for two days followed by 30% sucrose in PB until the brains sank completely. The brains were frozen and cut in transverse sections at 30 µm on a sliding freezing microtome. Every 200 µm tissues were mounted on slides and stained with cresyl violet. All slides were dehydrated with an ethanol series, cleared with Citrisolv (Fisher Scientific, Fair Lawn, N.J.) and coverslipped with Permount (Fisher Scientific, Fair Lawn, N.J.) for lesion site/size analysis.

For cohort 2, animals and their brains were processed identically to cohort 1 at six weeks following TBI. Free floating sections were washed three times for five minutes in PBS containing 0.1% Tween 20 (PBST) (Sigma). For Aβ and MAP2 staining, brain sections were blocked for 60 minutes at room temperature with 5% normal goat serum (Invitrogen, CA) in PBST. Sections were then incubated overnight at 4°C with rabbit polyclonal anti-Aβ 1-14 (1∶100; Abcam, ab2539) and mouse monoclonal anti-MAP2 (1∶100; Abcam ab11267), with 5% normal goat serum. The rabbit anti-Aβ antibody used in this study identifies amino acid residues 1–14 of Aβ and stains extracellular aggregates of Aβ peptides. MAP2, or Microtubule-associated protein 2, typically used as a marker for neurons, is a neuronal phosphoprotein that regulates the structure and stability of microtubules, neuronal morphogenesis, cytoskeleton dynamics and organelle trafficking in axons and dendrites.

The sections were washed five times for 10 minutes in PBST and then soaked in 5% normal goat serum, diluted in PBST, containing the corresponding secondary antibodies (goat anti-rabbit IgG-Alexa 488 (green) and goat anti-mouse IgG-Alexa 594 (red) (1∶500; Invitrogen) for 90 minutes. Finally, sections were washed five times for 10 minutes in PBST and three times for five minutes in PBS, then processed for Hoechst 33258 (bisBenzimideH 33258 trihydrochloride, Sigma) for 30 minutes, washed in PBS, then cover-slipped with Fluoromount (Sigma).

### Histological analyses

For cohort 1, the lesion sites were microscopically confirmed using a macroscope (Wild M420 and Nikon SMZ 1500) and a microscope (Nikon Microphot FX). Images of sections with the interval of 400 µm were traced using a camera lucida method. Cortical, hippocampal, and overall lesion volumes were determined by calculating the total volumes of neuronal loss in the injured hemisphere compared to the contralateral side.

For cohort 2, the number of extracellular Aβ deposits and MAP2 positive cells in the cortex and hippocampus were examined using an Olympus confocal microscope (Japan). Every sixth 30 µm-thick coronal tissue section was collected from each mouse. The number of extracellular Aβ deposits and MAP2 positive cells were counted in each of five high power fields (HPF) (200,000 µm^2^) and the averages were used for the statistical analyses. The regions of interest for estimation of extracellular Aβ deposition and MAP2 positive cells included the ccortex and the hippocampus, which were photographically captured (Axiophot2; Zeiss), and cells were quantified by counting per HPF view selected at random and corrected by the Abercrombie formula. Two captured fields in each coronal level, using three levels, were used to analyze the extracellular Aβ deposition and MAP2 positive cells in the cortex (AP, 1.5, 0.5, and −0.5 mm to bregma) and hippocampus (AP, −4.8, −5.3, and −5.8 mm) [22], using Scion Image software (Scion, Frederick, MD). Binary images were created using a distinct threshold, and then the positive areas were calculated [25]. Additionally, confocal analysis was performed using confocal microscope (Olympus).

### Statistical Analysis

All genotype/TBI groups were assessed statistically using IBM SPSS Statistics software. For the multiple post-TBI RAWM sessions, a 2×2 ANOVA was conducted with genotype (AD, NT) and TBI exposure (TBI, Sham) as the between-subjects factors. The errors of T4 and T5 were the dependent variables focused on to measure behavioral deficit. For the neurological assessment tasks, a 2×2 ANOVA (genotype x TBI exposure) was used to measure any group differences in the balance beam, and a Kruskal-Wallis test was used for the visual cliff task. Upon observing any significant differences between groups, subsequent t-tests were also conducted. All group comparisons having p<0.05 were considered significant.

## Results

### Lesion Volume

**Figure 2:**
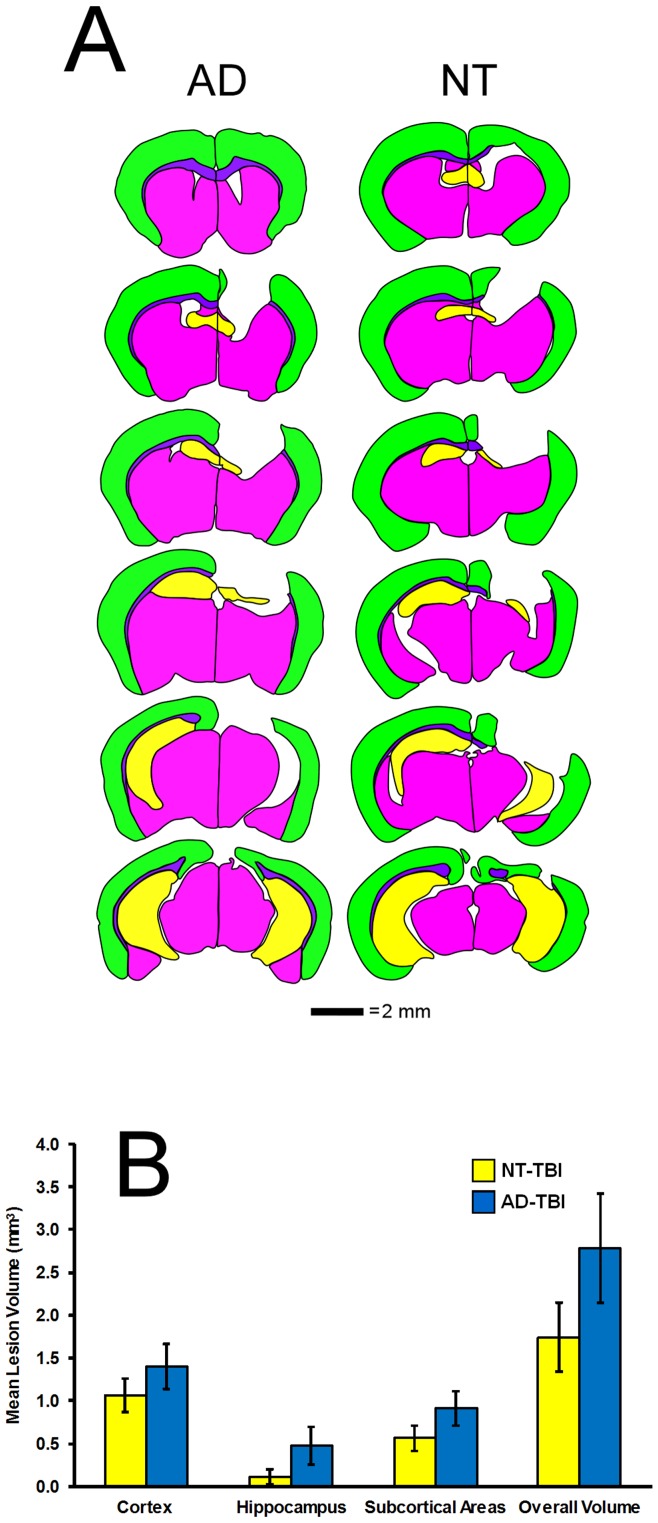
Two examples of lesion reconstructions for an AD- and NT-TBI mouse (A). Each figure is composed of transverse sections from anterior to posterior. Each section is color-coded to show the cortex (green), hippocampus (yellow), and subcortical areas (magenta). The lesion volumes for NT-TBI (yellow) and AD-TBI (blue) mice (**B**). There were no significant differences of lesion volume between NT-and AD-TBI mice. The error bars represent the SEM.

All lesions were made in the right hemisphere of TBI animals. Figure 2A shows two examples, in which damage was observed in the cortex, hippocampus, and subcortical structures to some degree. For cohort 1, there were no significant differences of lesion volume between AD- and NT-TBI mice, for the cortex, hippocampal area, subcortical area or overall lesion volume (p's > 0.05). In addition, there was no detectable reduction in brain volume of non-lesioned AD mice compared to non-lesioned NT mice. Figure 2B shows the mean lesion volumes of these structures.

### Pre-TBI RAWM Performance

**Figure 3:**
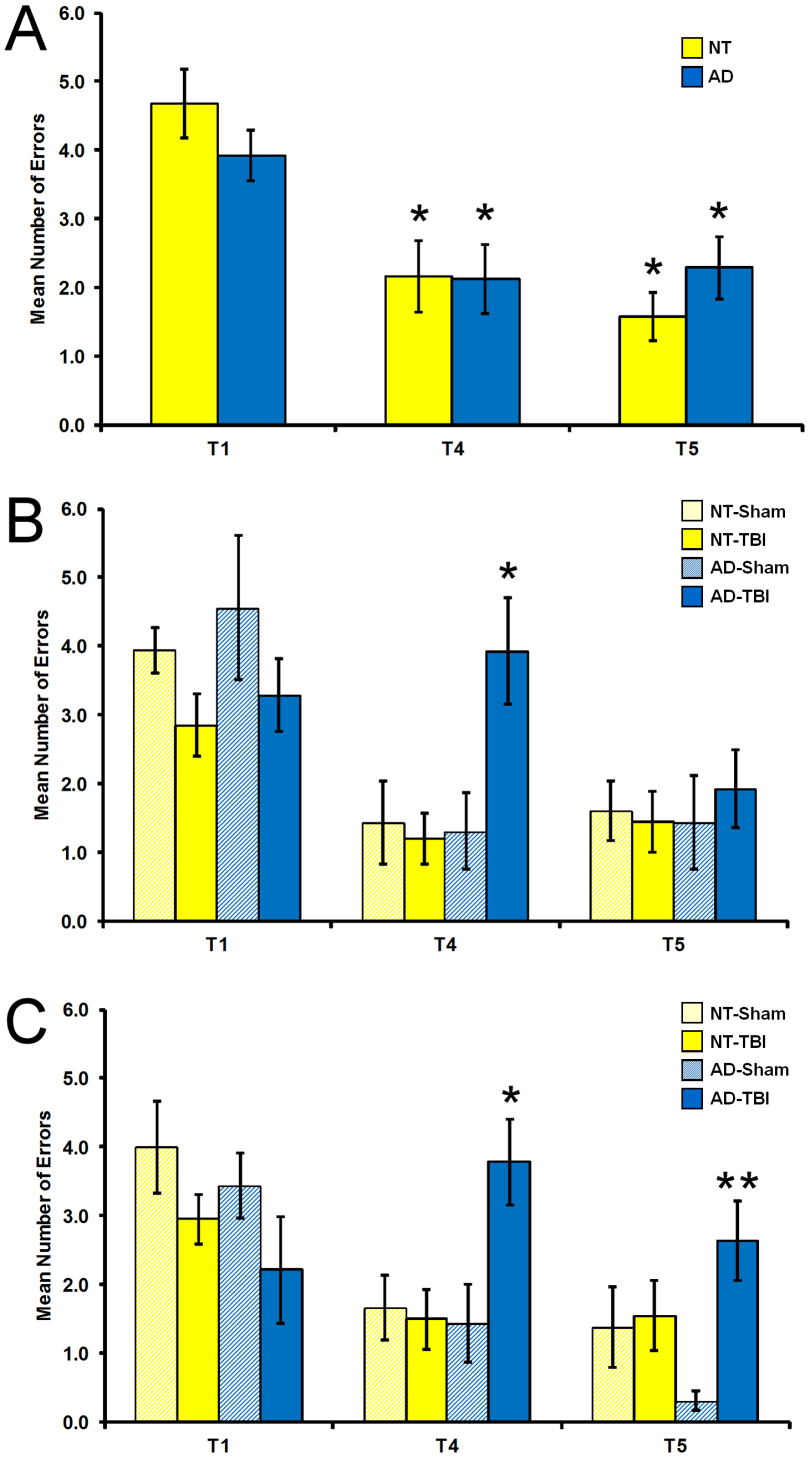
Prior to TBI surgery (A), the number of errors on trial 1 (T1), trial 4 (T4), and the 30 minute retention trial (T5) for NT (yellow) and AD (blue) mice during the final two days of RAWM testing. Asterisk indicates significant improvement from T1 for that group at p<0.05 or higher level of significance. For Post-TBI testing, the number of errors on T1, T4, T5 for the RAWM at two weeks (**B**) and six weeks (**C**) after TBI. NT-Sham (hatched yellow, n = 9), NT-TBI (solid yellow, n = 10), AD-Sham (hatched blue, n = 8), and AD-TBI (solid blue, n = 7) mice. Single asterisk indicates the AD-TBI group is significantly different from all other groups at p<0.02. Double asterisk indicates the AD-TBI group is significantly different from AD-Sham at p<0.01. The error bars represent the SEM.

Before TBI was performed, 19 AD and 19 NT mice were trained to perform in the RAWM over a 14 day period. Compared to their performance during naïve trial 1 (T1) of the final two-day block, AD and NT groups both were able to significantly improve performance during trial 4 (T4) and trial 5 (T5) (Figure 3A). Mice in both AD and NT groups performed at an average of around two or fewer errors across T4 and T5 during this final two-day block. Thus, AD and NT groups performed similarly in their learning (T4) and memory retain (T5) prior to surgery.

Animals in each genotype were then divided into the TBI and sham lesion groups to counterbalance learning performance. The mean number of errors were compared for NT-Sham (n = 9), NT-TBI (n = 10), AD-Sham (n = 9), and AD-TBI (n = 10) mice. A 2×2 Factorial ANOVA was conducted with genotype (NT, AD) and planned surgical treatment (TBI, Sham) for T1, T4 and T5. For T1, T4, and T5 there were no significant effects for genotype, treatment, or interaction (p's > 0.05).

### Post-TBI RAWM Performance

For the RAWM task, two weeks after TBI exposure, there were no significant effects in T1 for genotype, TBI exposure, or interaction of genotype by TBI exposure (p's > 0.05), indicating that all groups had the same random performance on this first trial of daily testing. For T4, genotype (F_1,33_ = 5.906, p = .021), TBI exposure (F_1,33_ = 4.927, p = .034), and a genotype by TBI exposure interaction (F_1,33_ = 7.167, p = .012), all supported the significant effect of impact of TBI on AD mice performance (p<0.02; asterisk on Figure 3B). TBI had no effect on performance of NT mice during T4 (p > 0.05). In contrast, TBI induced impairement in performance in AD mice during the same trial compared to AD-Sham mice, as indicated by their sharply higher number of errors during this trial (t_13_ = 2.989, p = .01). However, in T5, AD-TBI mice performed similar to controls; there was no main effect for genotype, TBI exposure, or interaction of genotype by TBI exposure (p's > 0.05).

Six weeks after TBI exposure (Figure 3C), there were significant effects for TBI exposure in T1 (F_1,33_ = 4.535, p = .042). During this first trial, TBI mice made more errors than mice without TBI (t_32_ = 2.386, p = .023), but there were no significant effects for genotype (p > 0.05). For T4, there was a main effect in terms of genotype (F_1,33_ = 4.441, p = .044), TBI exposure (F_1,33_ = 4.998, p = .033), and interaction of genotype by TBI exposure (F_1,33_ = 6.641, p = .015). The AD-TBI mice made significantly more errors than all other groups (p<0.02; asterisk on Figure 3C): AD-Sham mice (t_13_ = 2.979, p = .011); NT-TBI (t_15_ = 3.311, p = .005); NT-Sham (t_14_ = 2.977, p = .010). For T5, there were no significant differences between AD and NT groups (p > 0.05). However, there were significant effects for TBI exposure (F_1,33_ = 6.950, p = .013) and the interaction of genotype by TBI exposure (F_1,33_ = 5.269, p = .029). Specifically, AD-TBI mice made more errors than AD-Sham mice (t_13_ = 4.533, p = .001). In sharp contrast, NT-TBI mice had the same low level of T5 errors compared to NT-Sham mice (p > 0.05). However there were no differences between TBI groups (p > 0.05). Moreover, all groups were able to significantly improve their performance except for the AD-TBI group, across trials T1 vs. T4 and T1 vs. T5 (p's > 0.05), thus indicating a cognitive impairment specific for the AD-TBI group at this six week time point.

Concerning neurological evaluation (data not shown), there were no group differences in the balance beam task for genotype, TBI exposure, or interaction of genotype by TBI exposure (p's > 0.05). For the visual cliff task, there were also no significant group differences (p > 0.05).

### TBI expedites extracellular Aβ aggregation in the cortex of AD-transgenic mice

**Figure 4:**
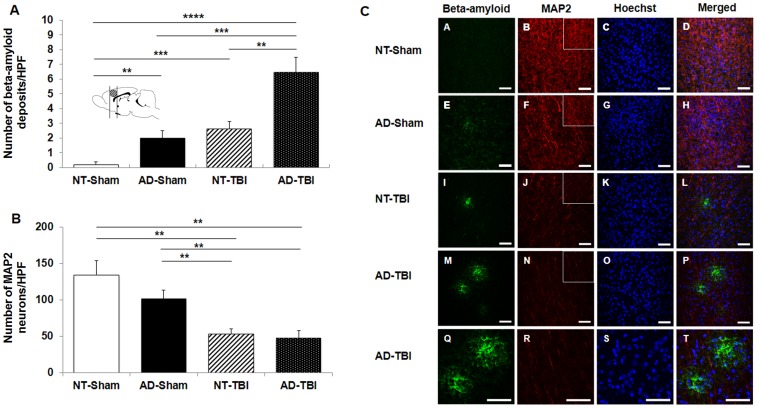
TBI accelerated extracellular Aβ deposits in the cortex of AD mice that received TBI. Statistical analysis revealed a significant increase in extracellular Aβ deposits in the cortex of AD mice that received TBI compared to the NT mice that received TBI and the AD and NT mice that underwent sham surgery (Panel A). Statistical analysis revealed that there was a significant decrease in the number of MAP2 positive cells in the cortex of AD-transgenic and NT mice that underwent TBI surgery compared to the AD and NT mice that received sham surgery (Panel B). Immunofluorescence for the detection of extracellular Aβ deposits and MAP2 (Panel C). Scale bars = 50 µm. The insets correspond to representative high magnifications of MAP2 images. Scale bars = 100 µm. The brain illustration shows the location of the brain slices chosen for histological analysis. ** p < 0.01, *** p < 0.001, **** p < 0.0001.

Aβ staining demonstrated that more extracellular Aβ deposits were found in the cortex of the AD-TBI group (Figure 4A, C). Of note, MAP2 positive cells showed decrease in the cortex of the AD-TBI group (Figure 4B, C). ANOVA revealed significant treatment effects on extracellular Aβ deposits in the cortex (F_3,69_ = 11.629, p<0.0001) (Figure 4A). Immunostaining revealed a significant increase in extracellular Aβ deposits in the cortex of the AD-TBI group (6.44±1.04 deposits/HPF) compared to the NT-TBI group (2.60±0.52 deposits/HPF, p<0.01), AD-Sham group (2.00±0.49 deposits/HPF, p<0.001) and NT-Sham group (0.20±0.20 deposits/HPF, p<0.0001) (Figure 4A, C). Along these same lines, the NT-TBI group also exhibited a significant increase in extracellular Aβ deposits in the cortex compared to the AD-Sham group (p<0.001) and NT-sham group (p<0.001) (Figure 4A). In addition, ANOVA detected significant treatment effects on MAP2 neuronal cell loss in the cortex (F_3,34_ = 11.553, p<0.0001). Of note, there was a significant decrease in MAP2 positive cells in the cortex of the AD-TBI group (47.20±11.00 neurons/ HPF) compared to the AD-Sham group (101.33±11.68 neurons/ HPF, p<0.01) and NT-Sham group (133.71±20.00 neurons/ HPF, p<0.01) (Figure 4B). Similarly, the NT-TBI group also exhibited a significant MAP2 neuronal cell loss in the cortex (53.13±7.19 neurons/ HPF) compared to the AD-Sham group (p<0.01) and NT-Sham group (p<0.01). There was no significant difference between AD-TBI and NT-TBI groups (p>0.05) (Figure 4B).

### TBI accelerates extracellular Aβ deposition in the hippocampus of AD-transgenic mice

**Figure 5:**
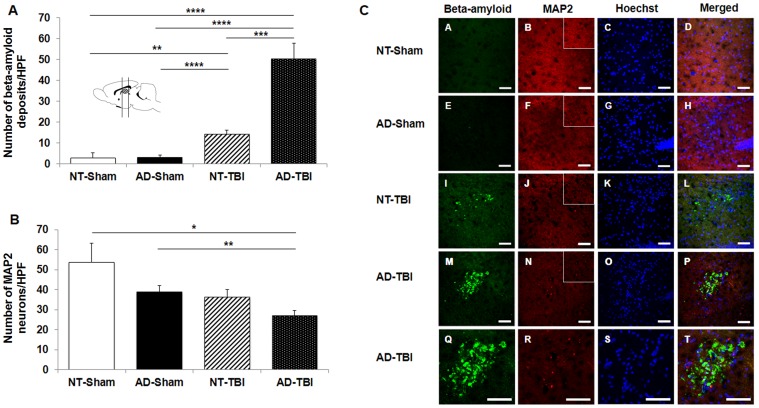
TBI accelerated extracellular Aβ deposits in the hippocampus of AD-TBI mice. Statistical analysis revealed a significant upregulation of extracellular Aβ deposits in the AD mice that received TBI compared to the NT mice that received TBI and the AD and NT mice that received sham surgery (Panel A). MAP2 staining revealed a significant decrease of MAP2 positive cells in the hippocampus of AD mice that received TBI compared to the AD and NT mice that received sham surgery (Panel B). Immunofluorescence for the detection of extracellular Aβ deposits and MAP2 (Panel C). Scale bars = 50 µm. The insets correspond to representative high magnifications of MAP2 images. Scale bars  = 100 µm. The brain illustration shows the location of the brain slices chosen for histological analysis. * p < 0.05, ** p < 0.01, *** p < 0.001, **** p < 0.0001.

Aβ staining demonstrated that more extracellular Aβ deposits were found in the hippocampus of the AD-TBI group (Figure 5A, C). Of note, MAP2 positive cells showed decrease in the hippocampus of the AD-TBI group (Figure 5B, C). ANOVA revealed significant treatment effects on the number of extracellular Aβ deposits in the hippocampus (F_3,57_ = 33.686, p<0.0001) (Figure 5A). There was a significant increase in the number of extracellular Aβ deposits in the hippocampus of the AD-TBI group (50.18±7.70 deposits/HPF), compared to those of the NT-TBI group (14.12±2.08 deposits/HPF, p<0.001), AD-Sham group (3.00±1.09 deposits/HPF, p<0.0001), and NT-Sham group (2.82±2.43 deposits/HPF, p<0.0001) (Figure 5A). Along these same lines, the NT-TBI group also exhibited a significant increase in extracellular Aβ deposits in the hippocampus compared to the AD-Sham group (p<0.0001) and NT-Sham group (p<0.01) (Figure 5A). In parallel, ANOVA revealed significant treatment effects on MAP2 neuronal cell loss in the hippocampus (F_3,42_ = 3.711, p<0.05). The number of MAP2 positive cells in the hippocampus was significantly lower in the AD-TBI group (27.00±2.53 neurons/HPF) compared to the AD-sham group (39.00±3.16 neurons/HPF, p<0.01) and NT-Sham group (53.81±9.49 neurons/HPF, p<0.05) (Figure 5B). Conversely, for the NT-TBI group (36.25±3.92 neurons/HPF) there was no significant difference in MAP2 positive cells when compared to the AD-Sham group (p>0.05) and NT-Sham group (p>0.05) (Figure 5B).

## Discussion

This study reports a causal role of TBI in AD-related pathological manisfestations. We demonstrate accelerated Aβ aggregations in the APP/PS1 transgenic mice at six weeks post-TBI accompanied by expedited cognitive impairment in presymptomatic AD mice. The recognition of this direct link between TBI and AD should aid in developing novel treatments directed at abrogating cellular injury and Aβ deposition in the TBI brain.

For cohort 1, AD mice with TBI made significantly more errors in the RAWM task than controls (i.e., AD mice without TBI and NT mice with or without TBI). Because there were no lesion volume differences between AD-TBI and NT-TBI mice, the deficits were unlikely to be due to the lesion size per se. Therefore, the results suggest that TBI precipitated cognitive deficits specifically in presymptomatic AD mice, but not NT mice. The deficits of AD-TBI mice were found in T4, but not T5, at two weeks post-TBI surgery, whereas the deficits were seen in both T4 and T5 at six weeks post-TBI. It is not clear why the behavioral effects in T5 were different between the two and six weeks post-TBI. However, one possibility is that TBI-induced extracellular Aβ deposits increased substantially between the two and six week behavioral test points. Another issue that warrants further studies is the significance of the morphological distortion of the brain structures caused by TBI. We noted that TBI resulted in changes of the shape and location of some brain structures, including the hippocampus. Although we measured the volume changes, we did not quantitatively analyze the gross morphological changes. Thus, it is possible that some axonal damage due to TBI was overlooked in our analyses.

The increase in extracellular Aβ deposits in the cortex and hippocampus of AD mice that received TBI surgery supports the notion that TBI induces the upregulation of enzymes activating the cleavage of amyloid precursor protein to Aβ [26]–[28] (Figure 4, 5). Although the histological damage induced by the TBI only directly affected the cortical region of the brain, extracellular Aβ deposition was detected throughout the cortex and the hippocampus of the AD mice. These results implicate an evolving cell death beyond the initial localized cortical damage induced by TBI. Vascular damage caused by TBI may play a critical role in the exacerbation of AD-like histopathology following TBI as seen in laboratory [29], [30] and clinical studies [29]–[33]. The breakdown of vasculature patency after TBI contributing to the acceleration of Aβ deposition in AD is thought to be mediated by a pro-inflammatory signaling pathway [32] which is a major pathological component of TBI [34], [35].

The results of this study have important clinical implications. Accelerated AD symptoms following TBI suggest that trauma is a major co-morbidity factor of AD. Of note, the risk of developing AD after severe TBI is significantly higher in individuals that lack the ApoEε4 allele [36]. The military population is at an increased risk of TBI, and accumulating evidence indicates similarly a trend of higher incidence of cognitive decline in soldiers with direct or even indirect exposure to TBI [37]. Accordingly, soldiers diagnosed with TBI, including mild TBI, may benefit from a regimen of AD medications to prevent the development of AD-like pathology.

In summary, we demonstrated that TBI expedites the presentation of AD-like cognitive deficits and histological pathology in transgenic mice. A recent paper [21] reported that the CCI TBI model promoted 3xTg-AD mice to display intra-axonal Aβ accumulations and increased phospho-tau immunoreactivity at 24 hours and up to seven days after TBI. The present study replicated the observed acceleration of Aβ aggregation [21], but also extended such AD pathology up to six weeks post-TBI. Our observed histological data should allow correlative analyses between extracellular Aβ deposits and cognitive impairments providing further support for a causal role of TBI in AD-related pathology. Finding novel treatments designed to limit cellular injury and reduce extracellular Aβ deposition in the brain may prove beneficial in AD and TBI.
